# Long-term transmission patterns and public health policies leading to malaria elimination in Panamá

**DOI:** 10.1186/s12936-020-03329-y

**Published:** 2020-07-23

**Authors:** Lisbeth Hurtado, Alberto Cumbrera, Chystrie Rigg, Milixa Perea, Ana María Santamaría, Luis Fernando Chaves, Dianik Moreno, Luis Romero, Jose Lasso, Lorenzo Caceres, Azael Saldaña, Jose E. Calzada

**Affiliations:** 1 Departamento de Análisis Epidemiológico y Bioestadísticas, Instituto Conmemorativo Gorgas de Estudios de la Salud, Panamá, República de Panamá; 2 Universidad de Panamá, Panamá, República de Panamá; 3 Dirección de Investigación y Desarrollo Tecnológico, Instituto Conmemorativo Gorgas de Estudios de la Salud, Panamá, República de Panamá; 4 Departamento de Investigación en Parasitología, Instituto Conmemorativo Gorgas de Estudios de la Salud, Panamá, República de Panamá; 5 Instituto Costarricense de Investigación Y Enseñanza en Nutrición Y Salud (INCIENSA), Tres Ríos, Cartago, Costa Rica; 6 Laboratorio Central de Referencia en Salud Publica, Instituto Conmemorativo Gorgas de Estudios de la Salud, Panamá, República de Panamá; 7 Departamento de Control de Vectores, Ministerio de Salud (MINSA), Panamá, República de Panamá; 8 Departamento de Investigación en Entomología Médica, Instituto Conmemorativo Gorgas de Estudios de la Salud, Panamá, República de Panamá

**Keywords:** Malaria elimination, *Plasmodium*, Epidemiology, Climate, Panamá

## Abstract

**Background:**

The present study provides a countrywide perspective of the malaria situation in Panamá over a long-term framework, with the purpose of identifying historical malaria resurgence events and their potential causes.

**Methods:**

A descriptive-ecological study was conducted by analysing demographic and epidemiological annual malaria time series data in Panamá (1884–2019) using several data sources. Malaria intensity indicators were calculated during the study period. The effects of El Niño Southern Oscillation on malaria transmission were also analysed using a retrospective analysis of malaria cases between 1957 and 2019.

**Results:**

Several factors were identified responsible for malaria resurgence in Panamá, mostly related with Malaria Control Programme weakening. During the past 20 years (2000–2019) malaria has progressively increased in prevalence within indigenous settlements, with a predominance of male cases and a high proportion (15% of total cases) in children less than 5 years old. During this period, a significant and increasing proportion of the *Plasmodium falciparum* cases were imported. Retrospective analysis (1957–2019) evidenced that ENSO had a significant impact on malaria transmission dynamics in Panamá.

**Conclusions:**

Data analysis confirmed that although authorities have been successful in focalizing malaria transmission in the country, there are still neglected issues to be solved and important intercultural barriers that need to be addressed in order to achieve elimination of the disease by 2022. This information will be useful for targeting strategies by the National Malaria Elimination Programme.

## Background

Panamá has a long history of tropical diseases control and research since the early twentieth century when arthropod vector-borne pathogens, such as yellow fever and malaria, posed great challenges to the construction of the Panamá Canal by the USA [1–5]. In fact, the French effort to construct a Canal through the Panamá isthmus was unsuccessful largely because of the failure to control vector-borne diseases that caused very high morbidity and mortality rates among the French Canal employees on the Isthmus [1, 6, 7]. At that time, the link between mosquitoes as vectors transmitting malaria and yellow fever had not yet been proven.[3, 6].

More than one century has passed since the opening of the Panamá Canal and despite the increasing economic development observed in the country during the past decade [8], malaria continues to be a major public health concern particularly affecting socially marginalized populations. Nearly 90% of the malaria cases registered in the country during the past 40 years are from poor indigenous regions, and around 1000 annual cases have been reported for the last 15 years, mostly (~ 94%) by *Plasmodium vivax* [9–12]; reflecting the neglected status of this disease and marked health inequities associated with ethnicity in Panamá [10, 11]. Indeed, the degree of socioeconomic inequity in Panamá reaches a value of 49.9 according to the Gini coefficient; one of the highest in Latin America [13].

During recent decades, malaria transmission intensity and infection risk had significant spatial and temporal fluctuations in Panamá. Currently, malaria transmission in Panamá is considered low, and most of the country is free of the disease. However, localized and well identified foci persist, characterized by a seasonal epidemic mainly due to *P. vivax* [12, 14]. Regarding malaria vectors, several species have been recorded in the Panamá, with *Anopheles albimanus* and *Anopheles punctimacula* being the most common and widely distributed across the country [15, 16].

The recent significant malaria transmission reduction observed recently in Panamá and neighbouring countries stimulated the launch of the initiative for Malaria Elimination in Mesoamerica. Aligned with this regional effort, Panamá, in coordination with the World Health Organization (WHO) and the Pan American Health Organization (PAHO), has revised its national strategy to eliminate malaria. As a result, the National Malaria Elimination Programme (NMEP) was launched in Panamá in May 2016 to finally eliminate local cases of malaria by 2020 and achieve full WHO certification by 2025 [12]. According to the NMEP, an initial and crucial step to optimize elimination strategies is the reassessment of the malaria situation in the country, as well as the analysis of biotic and abiotic factors that historically may have shaped transmission [12].

In this line, and to provide required information to fine tune interventions by the elimination programme, this study presents baseline data on long-term historical malaria transmission patterns in Panamá using historical records going back to 1884. The effects of significant climatic events, such as El Niño Southern Oscillation (ENSO), on malaria transmission using a retrospective analysis of malaria cases between 1957 and 2019 were also analysed. The overall purpose of this study is to contribute to the understanding of malaria dynamics in Panamá and to provide information to the NMEP for guiding malaria transmission reduction strategies that can lead to its elimination from Panamá.

## Methods

### Type of study

A descriptive ecological study was carried out using routinely collected malaria surveillance data. A preliminary temporal evaluation of malaria cases from 1884 to 2019 was conducted describing major environmental factors, historical events, public health policy changes and interventions that have influenced malaria transmission in Panamá. Subsequently, a thorough analysis was carried from 2000, the year in which the global roadmap to eliminate malaria was conceived, to 2019.

### Study site and population

**Fig. 1:**
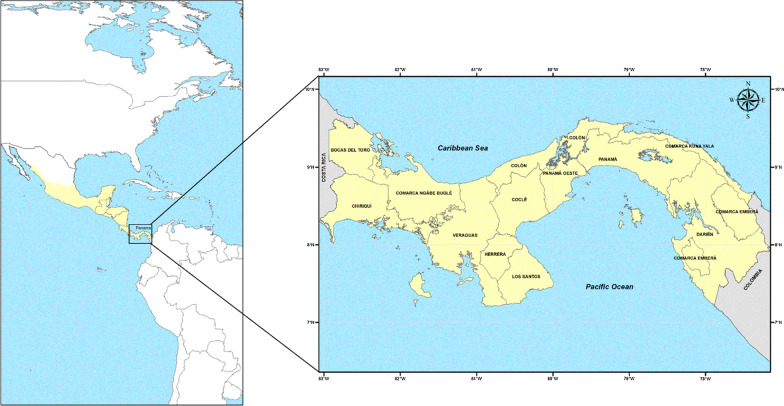
Map of the Americas showing location of Panamá and the Mesoamerica sub-region and map of Panamá showing location of provinces and indigenous regions (“Comarcas”)

Panamá is a narrow and highly biodiverse biogeographical corridor connecting South and Central America that lies between the Caribbean Sea to the north, and the Pacific Ocean to the south. It shares borders with Costa Rica (to the west) and Colombia (to the east); and has a total coastline that stretches over 2850 km (Fig. 1). It is one of the eight countries that conforms the Mesoamerican region (which includes Southeast Mexico and all the Central American nations), a region likely to eliminate malaria in a short period according to PAHO.

The country is administratively organized in ten provinces and five indigenous regions (called “Comarcas”) over a territorial extension of 75,517 km^2^, of which 16,598.6 km^2^ (22.0% of the Panamanian territory) belong to the following indigenous semi-autonomous Comarcas: East from the Panamá Canal, Guna Yala, Madungandí, Wargandí and Emberá Wounáan; and West of the Panamá Canal, Ngäbe Buglé (Fig. 1). Around 12.0% of the estimated population of the country (4,058,372 inhabitants by 2016) lives within these territories that were created in the Panamanian Constitution to provide full autonomy and social integration for Amerindian minority populations within the multicultural and ethnically.

diverse definition of Panamá. For conducting epidemiological surveillance, the Ministry of Health has established 16 Health Regions that in most cases correspond to the geopolitical provinces/comarcas of the country; except for the Province of Panamá that is subdivided into four regions: Panamá Metro, Panamá Este (East Panamá), Panamá Norte (North Panamá) and San Miguelito. The Darién Health Region includes the Darién province and the Comarcas Emberá-Wounaan and Guna Wargandí. Within each Health Region there is a Vector Control Department, where the National Malaria Control Programme (NMCP) operates. In Panama, the NMCP guides and coordinates all malaria control activities in the country, including treatment, sample-data collection and data analysis. Prevention and control activities are mainly focused on early diagnosis that relies on microscopy and on prompt treatment. Malaria cases are mostly detected by active surveillance performed by the National Malaria Control Programme (NMCP) personnel in all endemic areas. Indeed, more than 70% of the confirmed cases during 2012–2016 were detected through active surveillance. Health facilities conduct passive case detection without malaria-specific screening centres. These services are free of charge.

### Climate data

The climate and natural vegetation of Panamá are typically tropical. Humid tropical rainforest predominates with moderately high temperature (average 27.5 °C, minimum 21.5 °C and maximum 33.9 °C) stable throughout the year; and a high relative humidity (90%, minimum 88% and maximum 95%) due to the two large oceanic masses that converge on the isthmus. The country presents a unimodal rainfall pattern with abundant rainfall in most of the territory (range between 1000 and 5000 mm) during the rainy season that extends from May to November; and a dry season with almost total rain absence from December to April [17].

### Malaria climatic and demographic data sources

Malaria case records and datasets were obtained from the National Malaria Surveillance Database and the Weekly Epidemiological Reports prepared by the Ministry of Health from 2000 to 2019. Demographic information and geopolitical division of the country were obtained from the Dirección de de Estadística y Censo of Contraloría General of the Republic of Panamá [18]. Other malaria historical and epidemiological data used in this study were collected from published and grey literature. Grey literature included government malaria reports, technical reports, evaluations, policies and procedures of the Malaria Programme, and epidemiological data and maps generated by the NMCP from the Department of Vector Control of the Ministry of Health (MoH), and by the Instituto Conmemorativo Gorgas de Estudios de la Salud (ICGES) from 1930 to 2019. The data from 1884–1913 were based on clinical diagnostic and limited to the Panamá Canal area, while starting in1930 all data were based on microscopic blood slide examination.

For the temporal analysis of the annual malaria case data, years from 1957 to 2019 were classified in different ENSO phases following the classification by the United States National Oceanic and Atmospheric Administration Climate Prediction Center (https://www.cpc.ncep.noaa.gov/data/indices/ersst3b.nino.mth.81-10.ascii).

### Data analysis

Annual malaria incidence per 1000 was estimated as the ratio between the number of malaria cases and population size estimates for each year, multiplied by 1000. Annual Parasitic Incidence (API) from 1957 to 2018 were calculated considering the number of malaria cases among the population at risk of the respective year per 100,000 inhabitants. The population at risk was obtained from the estimated population for each year. The variables evaluated over time for positive cases were gender, age, locality of infection, malaria species and *Plasmodium* spp. imported cases based on travel history. Routinely, all positive slides and 10% of the negative slides, are confirmed by the Public Health Central Reference Laboratory at ICGES, following the national guidelines for malaria control of the MoH of Panamá [19].

A Chi square test and Z-test were used to demographically compare malaria case rates by gender and age groups. To understand the evolution of malaria, the incidence was analysed according to the origin of the malaria infection. For comparison purposes, the rates between the Health Regions were adjusted by the direct method of standardization, using the standard population that corresponded to the 2010 census. This information was depicted by cartography for target years that were at that time designated to comply with the malaria elimination goals (2000, 2010 and 2015), together with malaria epidemic years (2005 and 2012) and the current situation (2019) towards the 2020 objective.

A regression analysis was used to study the series of malaria cases and the ENSO phases from 1957 to 2019. Following this purpose, years were classified according to the occurrence of ENSO events as: “warm” phase for years dominated by extremely high values in sea surface temperature 4 SST-4 anomalies; “cold” phase for years dominated by low SST-4 values, and “normal” for years without extreme activity.

To identify potential breakpoints, i.e., time points when the average number of malaria cases changed [10, 20, 21], in annual malaria cases, we plotted the annual difference in the number of cases and estimated the 2.5 and 97.5 quantiles of these annual changes distribution [22]. Values outside these extreme quantiles were tested as potential breakpoints by estimating the Akaike information criterion (AIC), a metric that trades-off model fit and parameter number [22], of a negative binomial model, provided malaria cases counts were over-dispersed [23], with different means for the segments defined by the breakpoints and a covariate for the ENSO phase [24–26].

### Software used for data analysis

Malaria cases were processed and analysed with the Epi Info TM version 7.2 program. Microsoft Excel 2013 program was used to calculate and adjust rates, and results were presented in frequency tables or graphs. For mapping we used the software ArcGIS version 10.6. All other analyses were performed the statistical language R version 3.6.1.

### Ethical approval

As required by national regulations, this study was registered at the Coordinación en Regulación de Investigación para Salud, Dirección General de Salud Pública, Ministerio de Salud (Assigned Number:1338). This research was considered by the Comité de Bioética de la Investigación del Instituto Conmemorativo Gorgas de Estudios de la Salud and deemed exempt (N^o^113/CBI/ICGES/20). The search of suspected cases and all the diagnostic, treatment and documentation procedures of all malaria cases (detected via active and passive search) were conducted by the NMCP and local health centres’ technical personnel as part of the routine surveillance system for malaria control. Epidemiological information was also obtained from the NMCP databases. The confidentiality of the study subjects with malaria was protected and individual data were not shared.

## Results

### Pre-US Canal period (1884–1904)

**Fig. 2:**
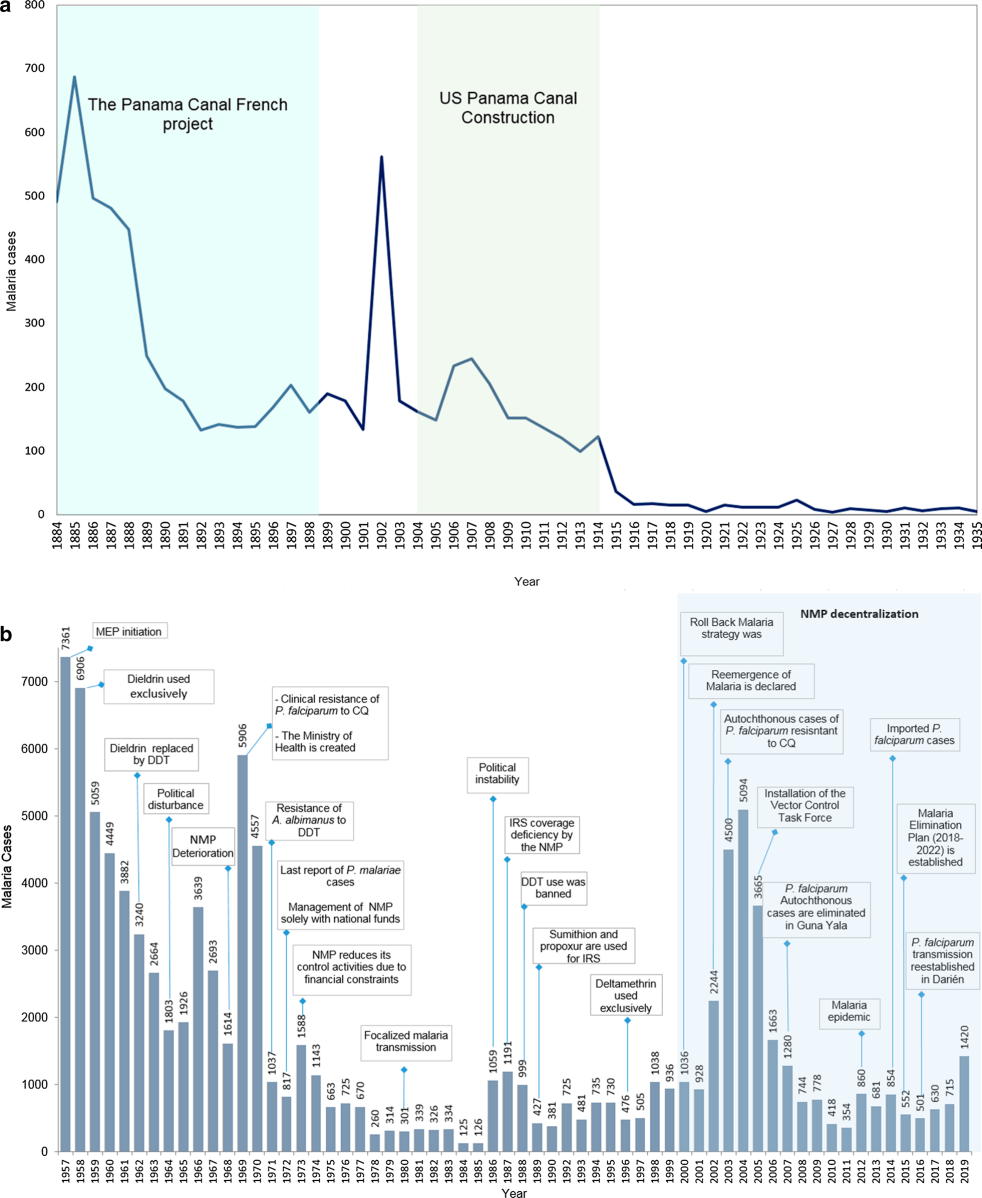
Evolution of annual malaria cases in Panama (**a**) from 1884 to 1935 (**b**) Timeline of reported malaria cases and major events in Panama, 1957–2019

Despite the excellent system of hospitals and patient overall healthcare, high mortalities rates attributed to malaria were observed among French Canal employees during the unsuccessful attempt of the French companies to build a Panamá Canal between 1881 and 1889 (Fig. 2a). Indeed, during the French development of the Panamá Canal a significant decline in the number of malaria cases was observed. However, efforts to control the disease during this period were highly ineffective due to the lack of information concerning malaria parasite transmission biology, particularly its transmission mode via mosquito bites. The general acceptance of the discovery proving that malaria was transmitted by mosquitoes—precisely when US took over the construction of the Panamá Canal in 1904—had profound influence on the incidence and distribution of malaria in Panamá and the rest of the endemic countries [3–7].

### 1905–1956 period

Since the beginning of the Panamá Canal construction by US in 1904, and thanks to the leadership and mosquito-environmental sanitation strategy designed and “militarily” executed by colonel and physician William Gorgas. In a short time, Panamá achieved a significant decrease in malaria mortality and morbidity in the two most important cities of the country at the end of the Canal: Panamá City and Colon (Fig. 2a), and small areas where the Canal construction was planned. The strictly enforced integrated programme involved mosquito larvae and adult control by activities such as drainage, brush and grass cutting, oiling, larviciding, screening and killing of adult mosquitoes inside houses. In addition, prophylactic quinine was provided freely to all workers along the Canal construction line [7]. The death rate due to malaria in the Canal employees dropped from 11.59 per 1000 in 1906 to 1.23 per 1000 in 1909.[6, 7]. Moreover, hospitalizations of Canal workers due to malaria gradually decreased from 82% in 1906 to 8% in 1913 [27]. Nevertheless, malaria control continued to be a challenge throughout the entire canal construction programme. During that time, for logistical and economic reasons reduced efforts were made to control the disease in the rest of the country, particularly outside the “influence area” of this engineering work [4–6]. Consequently, between 1905 and 1931 the disease was considered prevalent throughout the country. However, besides the well-kept statistical records in Panamanian towns were US corporations of commercial interest were functioning at that time (such as the United Fruit Company), data regarding the true prevalence and distribution of malaria in the entire country is incomplete [6].

In 1931, with the financial help of various organization as United Fruit Company, Gorgas Memorial Institute and the Rockefeller Foundation, a strong sanitation campaign based on pyrethroid fumigation and drainage of stagnant waters, was performed in banana plantation towns from the interior of the country; achieving a notable decrease in the disease in these selected regions, but far from the results observed in sanitized areas of the Canal Zone [6]. It was estimated that by 1947 malaria morbidity in the Panamanian population was around 8441 patients, and that before the beginning of the Global Malaria Elimination Programme in 1956, 2849 malaria cases were counted [28]. However, these figures should be viewed with caution because reporting of malaria cases and deaths was not mandatory in the country until 1957.

Between 1931 and 1949 the predominant species causing malaria in Panamá was *P. falciparum* [29–31]. In a study conducted by Clark and Komp (1938) it was found by microscopic assessment that *Plasmodium falciparum* infections reached 73.2%, *Plasmodium vivax* 13.9%, *Plasmodium malariae* 1.5%, mixed infections 9.7%. Mixed infections consisted mostly of *P. vivax* with *P. falciparum* [30]. It was noted, however, that with the introduction of DDT (Dichloro-Diphenyl Tricloethane) in the country for mosquito control in 1947, co-infections and re-infections with *P. falciparum* gradually disappeared, with a concomitant increase in cases due to *P. vivax* [28]. This change was most likely due to the distinct characteristics of the biology of *P. vivax* and the behaviour of its insect vector, which makes vivax malaria difficult to control. Among these specific features, the presence of *P. vivax* hypnozoites which can reactivate weeks or months after the primary infection. This is one of the major reasons of the predominance of this species after indoor residual spraying (IRS) was installed in the country.

During this period (1905–1956) important discoveries regarding methods for malaria control and treatment were developed and evaluated on the isthmus in seminal longitudinal studies carried out in endemic communities from Panamá [27, 29–42].

### 1957–1999 period

Historical trends of reported malaria cases and the most relevant events that have influenced malaria dynamics in Panamá between 1957–2019 are summarized in Fig. 2b. In 1956, following WHO and PAHO guidelines, the Global Malaria Eradication Programme (MEP) was launched in Panamá along with the rest of Central America and Mexico. That next year the official registration and the mandatory notification of malaria cases in the country also began. The Programme at that time operated under a vertical structure and was nationally disaggregated in operational areas [43, 44]. The first report in 1957 accounted for 7361 malaria cases mostly by *P. vivax*, 186 deaths and an API of 8.1 per thousand inhabitants (Fig. 2b, Additional file 1: Figure S1 and Additional file 2: Figure S2).

In the years following the MEP there was a marked decline in malaria cases, although evaluations carried out in 1960 proved that transmission remained active in all the country provinces [45]. In 1958, dieldrin was the insecticide solely used for IRS throughout the country, with annual periodicity cycles. In 1962, dieldrin was replaced by DDT with semiannual cycles per year, obtaining great success in the campaign [45, 46]. This change in insecticide was for economical and logistical reasons, not because of an evidenced *Anopheles* resistance to dieldrin in the country. Furthermore, dieldrin was considered a highly toxic insecticide and there was reluctance to its application because, according to the residents, it killed their domestic animals. There were also reports of *An. albimanus* resistance to dieldrin in El Salvador [46, 47].

Within the next few years following the eradication campaign, the number of cases continued to drop, but not to the figures expected by the MEP [45]. This decline trend in morbidity was observed until 1966, when malaria reached alarming values, reaching 3639 cases and an API of 3.0 (Fig. 2b, Additional file 1: Figure S1 and Additional file 2: Figure S2).

With the creation of the MoH in 1969, the progress of the Elimination Programme became one of the priorities of the national health authorities. However, the statistical data for that year were discouraging; malaria incidence raised to 5906 cases with 24 deaths [47]. Moreover, the API reached 4.4 per thousand inhabitants, the highest since 1960 and the greatest magnitude observed to the present (Fig. 2b, Additional file 1: Figure S1 and Additional file 2: Figure S2). Around 90% of the cases registered that year where from the provinces of Panamá, Colón and Darién (Fig. 1). Field studies carried out in 1970 confirmed for the first-time clinical resistance of *P. falciparum* to chloroquine (CQ) in some locations from Panamá Province [48]. Thus, CQ was replaced by sulfadimethoxine associated with pyrimethamine and used for radical treatment in those areas. In general, the period between 1957–1970 was marked by political disturbance and deterioration of the MEP. Thus, it was the period with greatest morbidity and mortality observed in the history of malaria in Panamá (Fig. 2b).

Between 1971 and 1972 resistance of *An. albimanus* to DDT was detected in various regions of the country and DDT was replaced by the carbamate Propoxur for IRS in areas where DDT resistance was verified [52, 53]. By 1972, the last report of *P. malariae* infection occurred [54], with no further cases reported to date. The following year (1973) international funding for the MEP concluded and thus the programme started to be exclusively financed by national funds. Consequently, in that same year there were difficulties in materials supplies, deficiencies in supervision and in implementing control activities in remote areas [51]; a situation that promoted malaria epidemics in several regions of the country totaling 1588 cases (Fig. 2b). The most affected areas were in the eastern provinces: Darién and San Blas (now known as Comarca Guna Yala), near the Colombian border (Fig. 1).

The period from 1975 to 1985 represented the lowest malaria incidence in Panamá since the MEP was created in 1957, with a focalized transmission of malaria (Fig. 2b). However, in the late 1980s (1985–1989) Panamá went through a serious political crisis accompanied by an economic recession that culminated in the military invasion by USA in 1989 [10]. This crisis had a profound effect in the NMCP activities. There were deficiencies to cover all expenses, mainly for the purchase of insecticides and to cover operating costs that would allow adequate IRS coverage [48]. The effects were rapidly felt on the declining case trend observed during the previous years (Fig. 2b). Between 1986–1988 an annual average of 1000 cases was registered, mainly due to *P. vivax* epidemics in indigenous remote communities from the Province of Darién and the Eastern region from Panamá province, accounting up to 95.0% of the total cases registered in the country during that period. Given vector resistance and the attributed detrimental effects to health, in 1988 the use of DDT for vector control was banned and replaced by carbamate (Sumithion 40% WP or Sumithion 50% EC depending on the household physical characteristics) and organophosphate (Propoxur) insecticides. In 1993, pyrethroids (Cyfluthrin, Solfac Deltamethrin, and K-othrine) were evaluated, but at that time were not implemented by the NMP as alternatives for IRS [49]. In 1996, deltamethrin was reevaluated and that same year it began to be used, replacing Sumithion. This change represented important savings for the NMP since deltamethrin was applied twice per year whilst Sumithion cycle was 3 times a year. However, deltamethrin was discontinued in 2002 and replaced by fenitrotion, after the resistance of *An. albimanus* to this insecticide was detected.

In the period between 1990–2000 the epidemiological pattern varied from year to year, with an average number of cases 725 ± 161 and an API in the range from 0.2 to 0.4 per 1000 inhabitants (Fig. 2b, Additional file 1: Figure S1 and Additional file 2: Figure S2).

### 2000–2019 period

By the year 2000, malaria morbidity rate reached 36.5 per thousand inhabitants in the country and *P. viva*x was responsible for 96% of infections. Two important issues regarding malaria control took place in Panamá at the beginning of the millennium. First, the country joined the Rollback Malaria (RBM) strategy proposed by the WHO, that focused more in control than elimination of the disease [50]. Second, following international guidelines the MoH completed the process of decentralization of the malaria programme. 3 years later, in 2002, the malaria reemergence was declared in Panamá with 2244 cases and an incidence of 75.7 per 100,000 (Fig. 2b). This number represented a 2.4-fold higher relative risk compared with the incidence observed in 2001, the previous year. Furthermore, it was the highest incidence in the last 27 years, only comparable with the one observed in 1974 (73 per 100,000). Not only malaria risk increased in 2002, but also the disease significantly spread throughout the country. Making things worse, in 2003 autochthonous *P. falciparum* transmission resumed in Kuna Yala and Eastern Panamá, a situation not observed since 1970. It was also observed that circulating *P. falciparum* parasites in Panamá presented mutations that conferred resistance to chloroquine and partial resistance to antifolates, precisely the first and second line anti-malarial drugs used to treat *P. falciparum* cases by the NMP at that time [55, 56]. These relevant resistance findings were later confirmed using molecular barcode assays developed for *P. falciparum* [57].

The situation continued to deteriorate reaching a peak of 5094 malaria cases and 6 officially recorded deaths in 2004; figures only comparable to what occurred in the country in the late 1960s (Fig. 2b).

To tackle this public health crisis a Vector Control Task Group was created by the MoH. For this purpose, a crisis budget was allocated to this Group to be exclusively used for malaria control activities, without intromission by any other entities from the administrative structure. All staff and resources of the programme were placed under the coordination of the programme supervisor and an intensive operational plan was established to guarantee the following activities in remote areas: an active surveillance, rapid outbreak containment and a high IRS coverage. In this way, only by means of administrative modifications without changes in the attention guidelines, four months after the Vector Control Task Group creation a significant drop in the incidence rate was observed (Fig. 2b). At the end of 2005, the API was 1.4, a value that represented a 29% decrease compare with the previous year (API = 1.7). This decreasing trend continued with a 70% reduction (API = 0.5) in 2006 and a 76% reduction (API = 0.4) in 2007, reaching 354 cases and an API = 0.1 in 2011; the lowest incidence since 1985 (Additional file 2: Figure S2). Additionally, the autochthonous transmission of *P. falciparum* was eliminated in Guna Yala and the mortality rate significantly decreased.

However, in 2012 when the country was in a sustained control phase, a rebound in malaria transmission was observed, doubling the number of cases (354 and 860) from the previous year. Unfortunately, from 2013 to 2019 the number of malaria annual cases have remained above 500, reaching a peak of more than 1400 cases in 2019. More importantly, since 2015 *P. falciparum* transmission has re-emerged in eastern regions of the country.

Regarding regional strategies and commitments to eliminate malaria, Panamá did not meet the goal of 75% reduction of malaria morbidity at the end of 2014 set at the 58th World Health Assembly using as baseline estimated cases for 2000 [54]; and most likely, Panamá will not achieve the goal to eliminate local malaria cases by 2020 established by NMEP in 2016 [12].

### Malaria distribution by age, sex, and geographic location; 2000–2019

**Fig. 3:**
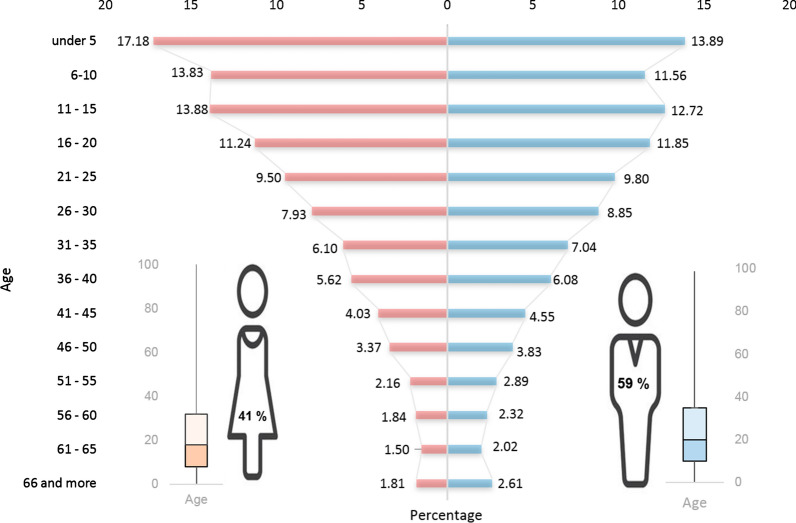
Malaria by Annual Parasitic Index distribution in Panamá between 2000 and 2018 per age group and by gender

**Fig. 4:**
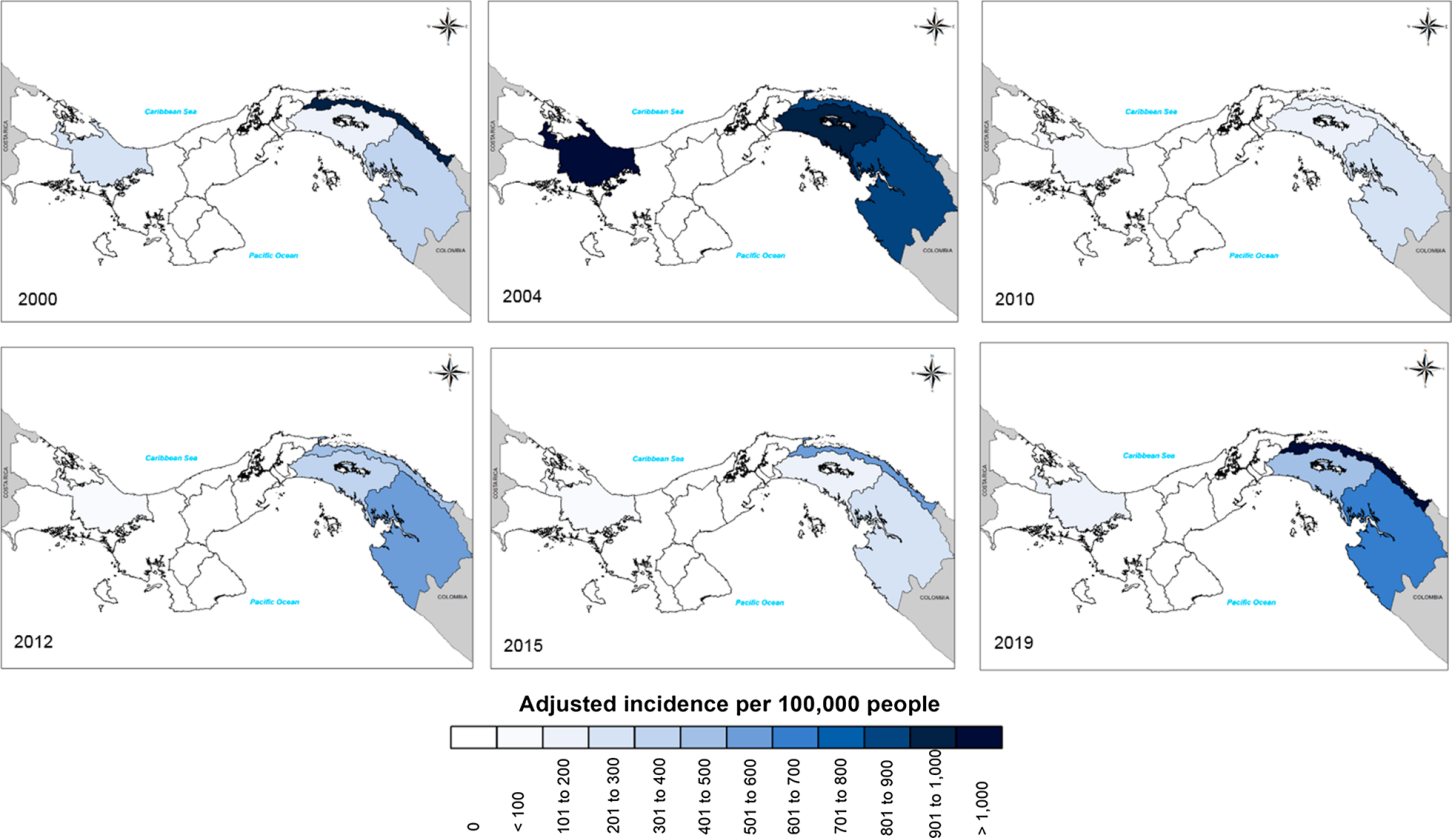
Malaria risk map of Panamá based on annual adjusted incidence rate: 2000, 2004, 2010, 2012, 2015 and 2019

In general, from 2000 to 2019, Panamá has accumulated a total of 28,921 infected people and 25 deaths from malaria. In this period morbidity has been more frequently observed in men than in women (58.8 vs 41.2%; *p* < 0.001). A significant difference was found when comparing malaria case among age groups (*p* < 0.001) (Fig. 3). Half of the infected population was 19 years old or younger, and 41% corresponded to children under fifteen years (9297/22,712), especially infants up to five years. In men, 75% of the cases occurred among those who were below 35 years of age, while in women the age was 32 years or less (Fig. 3). Additionally, malaria has progressively increased its prevalence within indigenous settlements. In 2005, 41.8% of the total malaria cases were from indigenous communities, while in 2016 this proportion reached 84.6% and in 2019 more than 90%. In fact, more than 70% of the cases accumulated in the country since 2005 come from indigenous communities located in the East of Panamá (Fig. 4).

### Imported malaria 2000–2019

**Fig. 5:**
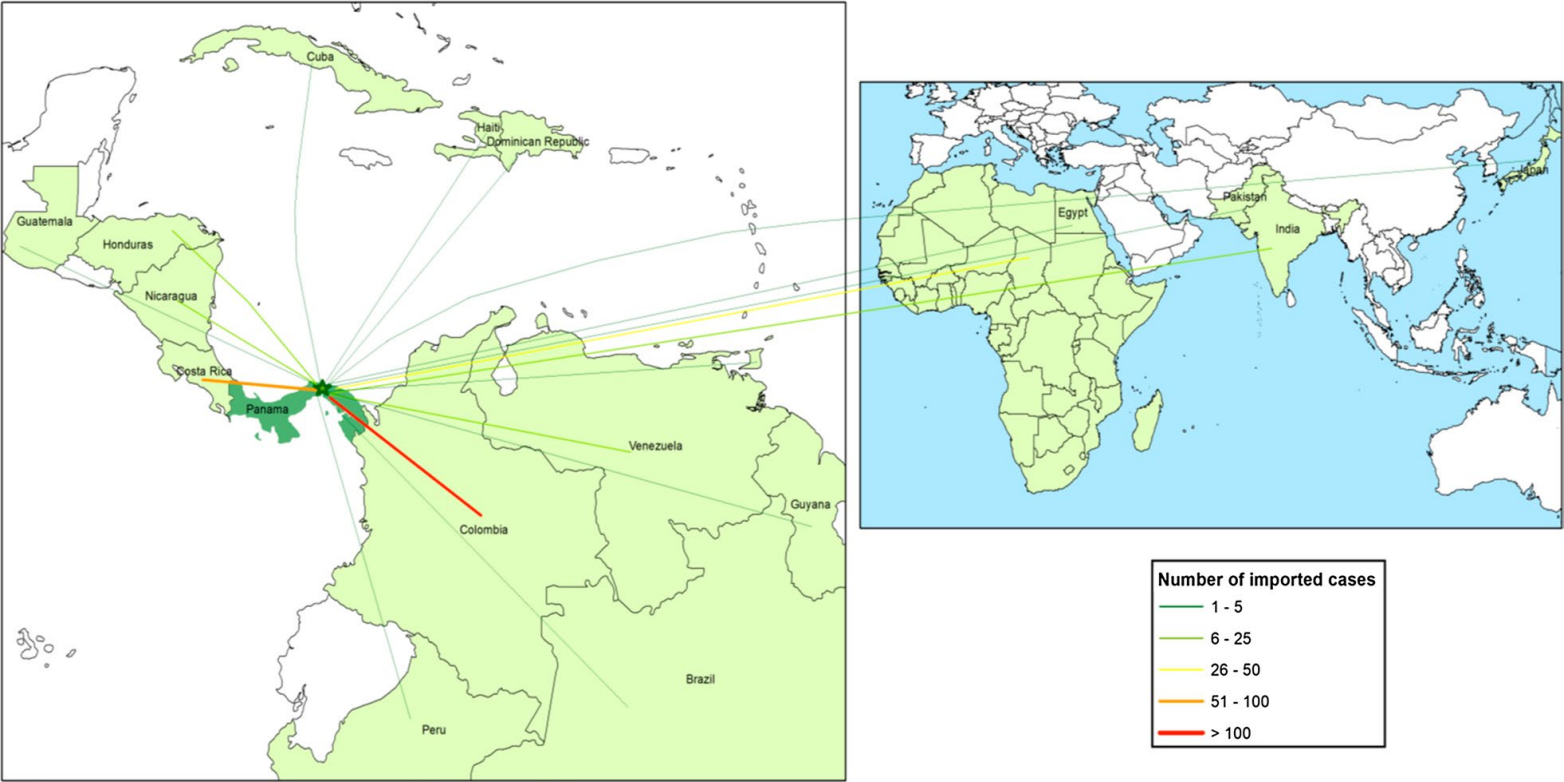
Malaria in Panamá by country from which cases were imported between 2000 and 2019

The Panamanian-Colombian border represents an important and continuous threat to accomplish the elimination goal set by the NMEP. Indeed, more than 14,000 migrants crossed into Panamá illegally from Colombia between January and June of 2019 [58]. Around 55% of these illegal immigrants came from the Caribbean, primarily from Haiti and Cuba, 25% from Africa, 19% from Asia and the rest from South America. Between 2000 and 2019, 361 malaria imported cases have arrived from different regions of the world. Most cases were from the American region 81.6% (294/360), particularly Colombia (48.6%; 175/360), Costa Rica (18.8%; 68/360), Nicaragua (3.8%; 14/360) and Venezuela (3.6%; 13/360). Countries from the African Region (13.3%; 48/360) and from Southern Eurasia (5.2%; 18/360) also contributed with a significant percentage of imported malaria in Panamanian territory (Fig. 5). Of the imported cases during this period, 28.5% (103/361) were *P. falciparum* and 71.5% (258/361) *P. vivax*. The burden of imported falciparum malaria originated mostly from Colombia (55.3%) and ten countries from the African continent (38.8%) (Fig. 5, Additional file 3: Figure S3 and Additional file 4: Table S1).

### Association between malaria incidence and ENSO events

**Fig. 6:**
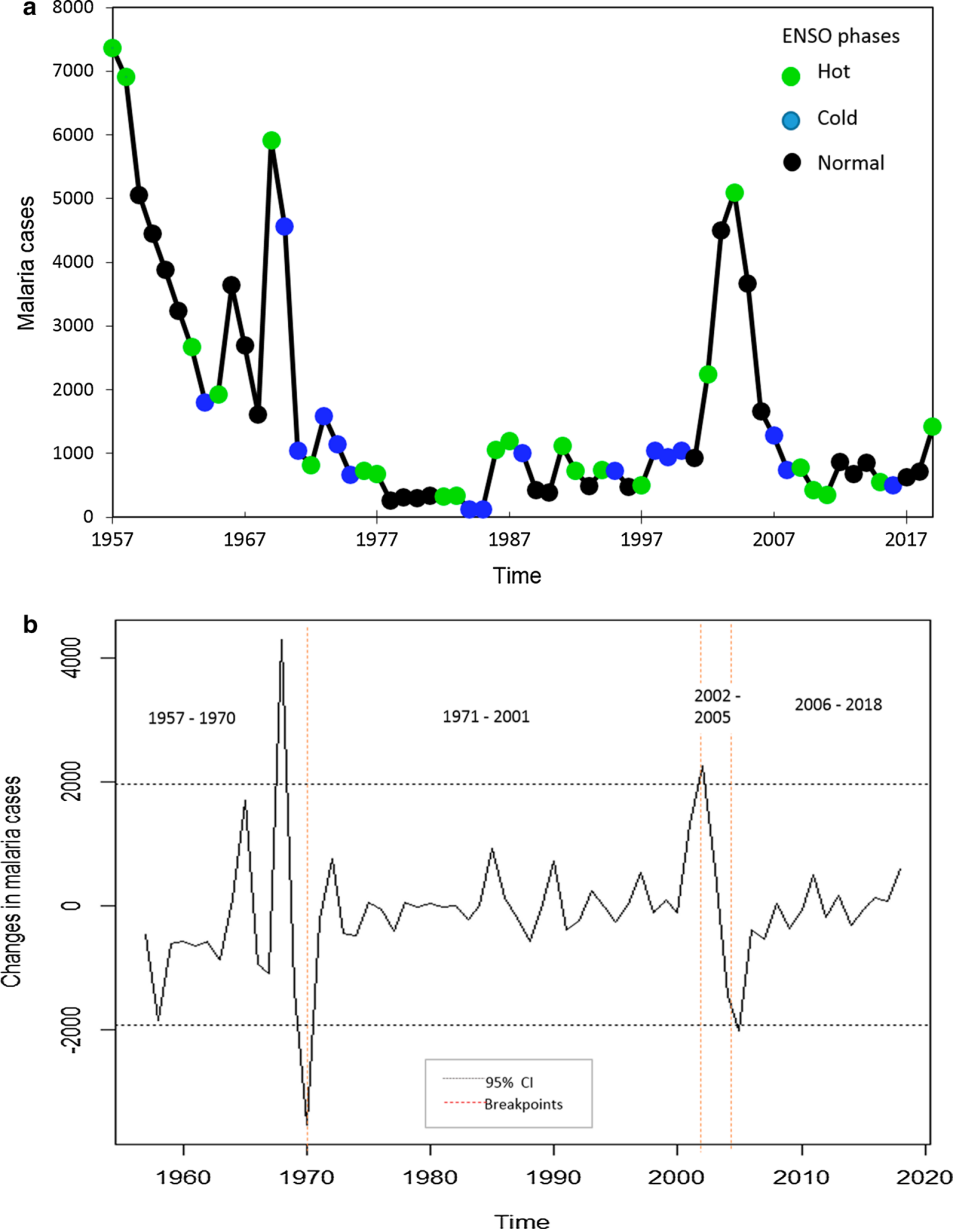
Malaria cases in Panamá and El Niño Southern Oscillation phases from 1957 to 2019, (**a**) Historical behavior (**b**) Annual changes. The dotted lines indicate the 2.5 and 97.5 quantiles of the changes distribution

Annual malaria cases recorded in Panamá between 1957–2019, with years classified according to different ENSO phases is shown in Fig. 6a. Figure 6b shows potential breakpoints in malaria transmission that occurred in 1968, 1970, 2002 and 2005. In Table 1, AIC values are indicated for models that split the time series in 5, 4 and 3 segments and for a null model without segments; the best model included the following 4-time segments 1957–1970,1971–2002,2003–2005,2006–2018. Parameter estimates for this best model are shown in Table 2. On average for the period 1957–1970 there were 3364 malaria cases per year during normal ENSO phase years, a number that increased by 37 and 38% during the Cold and Hot ENSO phases respectively. For the 1971–2002 period, the number of malaria cases significantly decreased by 83% when compared with 1957–1970 (P < 0.05), while for 2002–2005 it increased by 17% when compared with 1957–1970, although not significantly. This corresponds to the time when malaria transmission increased following the decentralization of the NMCP. From 2006 to 2018, the number of malaria cases significantly decreased by 79% when compared with 1957–1970 (P < 0.05), to a level similar to what was observed between 1971 and 2002.

**Table 1:** Akaike information criterion for negative binomial models explaining the number of malaria cases through different time segments

No. segments	Segments	AIC
5	1957–1968,1969–1970,1971–2002,2003–2005,2006–2018	988.3
4	1957–1970,1971–2002,2003–2005,2006–2018	986.5
4	1957–1968,1969–2002,2003–2005,2006–2018	1021.8
4	1957–1968,1969–1970,1971–2005,2006–2018	1028.3
4	1957–1968,1969–1970,1971–2002,2003–2018	1017.5
3	1957–2002,2003–2005,2006–2018	1055.0
3	1957–1970,1971–2005,2006–2018	1027.0
3	1957–1970,1971–2002,2003–2018	1015.7
1	1957–2018	1063.1

**Table 2:** Parameter estimates for the best negative binomial model explaining the number of malaria cases as function of the transmission time segment and ENSO phase

Parameter	Case number change	Estimate	Std. error	*z* value	Pr( >|z|)
ENSO-Normal 1957–1970	3364.38	8.12	0.15	54.15	< 2e−16*
1971–2001	0.17	− 1.77	0.16	− 10.77	< 2e−16*
2002–2005	1.17	0.16	0.32	0.50	0.62
2006–2018	0.21	− 1.58	0.19	− 8.32	< 2e−16*
ENSO-Cold	1.37	0.31	0.17	1.89	0.059
ENSO-Hot	1.38	0.32	0.15	2.19	0.028*
Overdispersion	3.98	0.68	–	–	–

*Statistically significant (*P* < 0.05)

## Discussion

Over the past decades, different malaria control strategies have been implemented in Panamá, most following guidelines from international agencies, and showing a spectrum of different effects regarding malaria transmission reduction. However, true political and financial commitment to malaria control and research have been cyclical, often curtailing successful efforts. The present study provides a countrywide perspective of the malaria situation in Panamá over a long-term framework, with the purpose of identifying historical malaria resurgence events, and their potential causes.

In this respect, the present study has suggested malaria reemergence has been unequivocally related with weakening the Malaria Control Programme, either following funding disruptions, administrative re-organization and other policy changes that form part of structural adjustment programs encouraged by multilateral financial institutions [59]. For instance, the dissolution of the global eradication campaign in 1969 and the subsequent political neglect of malaria, saw a huge decrease in mosquito control in Panamá investment, dropping from $1.20 per capita per year to just 19 cents [27, 60]. Similarly, the decentralization of the Malaria Programme, initiated in 1996, was fully implemented in 2000, and had profound effects. The poorly executed decentralization process resulted in a dramatic increase of malaria cases and the re-establishment of *P. falciparum* transmission in the eastern region of the country (Figs. 2b and 4). This situation is similar to what has been observed in much of Latin America, where malaria resurgence appears strongly correlated with diminished malaria programmes causing deficient IRS coverage [3, 61, 62], and more generally the disruption of other programmes aimed at eliminating structural determinants of malaria risk, for example housing improvement programmes [63] or universal health care [64]. Although technical problems including drug and insecticide resistance have also been described in Panamá (Fig. 2b) [55–57, 65]; the main causes of malaria resurgence in these events was not precisely the lack of technical solutions, but failures to promptly detect problems and to effectively implement technical solutions.

Panamá as part of the Mesoamerican region has committed to eliminate malaria local malaria transmission by 2020 and achieve full WHO certification by 2025 [12]. Results from this report, however, show that still there are important challenges that must be properly addressed in order to achieve elimination in Panamá, where elimination has not been achieved due to a lack of long-term commitment to tackle barriers to sustainably decrease malaria transmission to a pre-elimination level, as already done by El Salvador, Belize and Costa Rica in Mesoamerica [66–68]. In Panamá, the barriers to sustainably decrease malaria transmission reflect broader tensions faced from a culturally diverse society. For example, cultural barriers must be handled through the design and implementation of locally adapted and culturally sound intervention strategies consonant with the multiethnic characteristic of the country [69, 70]. It is well known that current western traditional strategies to control malaria are not well accepted by local indigenous populations. This is clearly illustrated by guna communities, where a disproportionate number of malaria cases (> 90% in 2019) occurs despite representing a relatively small proportion (around 2.4%) of the whole population [13, 18]. This crucial obstacle has been previously identified in several malaria studies and reports from indigenous communities in Panamá [9, 10, 71, 72], but remains a neglected issue to be solved. In this line, it is important to improve health services and reduced socioeconomical disparities in the indigenous reservations, precisely where malaria transmission persists [11, 14]. At present, poverty prevails in these rural inhabited by indigenous groups, where access to health and basic services is limited [11]. For example, there are 11 years less in life expectancy for native women and men living in indigenous territories compared with the overall population in Panamá (67.8 vs 79 years). Moreover, the maternal mortality rate is five times higher in Indigenous women who live in indigenous territories versus the national average for all women [73].

This analysis over a 20 year period (2000–2019) evidenced that when malaria cases were stratified by gender there was a significant predominance of males over females (58.8 vs 41.2%). A similar finding has been reported in many endemic countries of the region [74, 75]; possibly indicating that males are associated with higher exposure behaviours. A high and worrying proportion of malaria cases (15% of total cases) was observed in children less than 5 years old, suggesting that a significant amount of malaria transmission occurs within households. Furthermore, children under age five are most at risk for severe malaria due to low immunity. It is noteworthy that 53% of cases were among the economically active population between 16–55 years old; a situation that further increases the adversities of many indigenous families already impacted by marginalization, deprivation, and the threat of other health problems.

The continued risk of imported malaria cases also poses an enormous challenge politically, socially and logistically to malaria elimination in Panamá. Central America and particularly Panamá constitute a major transit area for mobile populations from all over the world towards the USA and Canada. For instance, more than 13,000 migrants crossed into Panamá illegally without malaria screening from Colombia during the first semester of 2019 [58]. According to official reports, around 55% of these migrants were from the Caribbean, primarily from Haiti and Cuba, 25% from Africa, 19% from Asia and 1% from South America [58, 76]. Mobile populations are more susceptible to malaria due to the nature of their migratory lifestyle. As described in this report, imported malaria cases, from both *P. falciparum* and *P. vivax*, have been confirmed in illegal migrants in Panamá. Indeed, many migrants are from countries where drug resistant strains circulate, a fact that continuously challenges the national malaria programme treatment policies [19]. In fact, recent *P. falciparum* outbreaks in areas where malaria transmission had been previously interrupted have been linked to migratory events across the Panamanian-Colombian border [9, 55, 56]. During this period (2000- 2019) a significant and increasing proportion of the *P. falciparum* cases were imported, mainly from Colombia (Additional file 3: Figure S3 and Additional file 4: Table S1). Moreover, in the last decade (2010–2019) more *P. falciparum* imported than autochthonous cases have been detected in the country (63 vs 45) (Additional file 3: Figure S3).

There is, therefore, an urgent need for an efficient cross-border cooperation particularly now when many Mesoamerican countries are involved in a malaria elimination campaign. An example that further describes this issue is the relatively high number of imported *P. falciparum* and *P. vivax* malaria cases (n = 34) from several African countries that were detected in Panama City—an urban area that has been free from autochthonous malaria for the last 60 years—during the “World Youth Day 2019”; a massive worldwide Catholic event that took place in January 2019.

Panamá also has unique location and geographic characteristics—an extended coastline and a narrow mainland—that renders the nation highly vulnerable to weather-related events. Here, evidence about ENSO impacts on malaria transmission dynamics in Panamá was added. Previously, it has been described that malaria transmission in the main endemic regions of Panamá, Comarca Guna Yala and Comarca Guna de Madugandi, peaks follow ENSO oscillations [10, 78], and are probably associated with increases in vector abundance. Understanding how and when ENSO impacts malaria transmission in a specific country and, more generally in a region like Mesoamerica, can be useful for preparing to timely deploy malaria control activities in Panamá.

Thus, several abiotic and biotic factors have been identified in this study most likely responsible for malaria resurgence in Panamá. However, this study has a number of limitations since the analyses have been based on annual data, thus limiting information about seasonal malaria transmission patterns. Data was collected from multiple sources. Early records were likely biased to the Panamá Canal zone and based on clinical assessments, while more recent numbers are from the whole country and based on microscopic slide examination. These differences did not allow the comparison of the different datasets, but the homogeneity of the recent years allowed the analysis considering the ENSO cycles. There were also some missing data concerning gender and age of malaria annual cases in databases held by the MoH. Regarding malaria imported cases, while WHO makes recommendations on the timeframe used to classifying malaria infections as imported, in some cases it was difficult to distinguish between local and imported malaria based solely on the travel history. Finally, little has been said about vector species [16], parasite genetic diversity [11] and other risk factors that might shape malaria transmission at an individual, not ecological, scale [79, 80].

The major obstacles to achieve malaria elimination in Panamá have already been highlighted in many studies and reports; the most important problems and their solutions already clearly understood from the time when W. C. Gorgas eliminated malaria from the Canal Zone. Now, a political commitment in key nationwide health directorates, under strong and courageous leadership, should secure and execute long-term funding to successfully consolidate the surveillance and health care systems needed to sustainably eliminate malaria from Panamá. In that sense, international and national funds designated for malaria control/elimination in the region must support strengthening vector-parasite control activities while also improving health infrastructure and services, particularly, in the already recognized malaria transmission hotspots of Panamá. During recent years a large proportion of Panamá`s malaria elimination funding has been diluted in the employment of international experts and financing regional meetings that, although necessary, are not able to reduce malaria transmission, and a low priority to achieve or accelerate malaria elimination from Panamá.

## Conclusions

During the Panamá Canal construction period and through the first half of the twentieth century, Panamá was considered a model country for the implementation of new malaria treatments and vector control measures [5, 6, 27, 81]. Nowadays, despite its relative small size and population and benefits, from a sustained economic development over the past decade [8]; the country struggles to eliminate malaria from all of its territory, facing financial and logistical constraints, reflecting the true neglected status of malaria in a country where more than 90% of malaria cases in the past 10 years have been reported in socially excluded indigenous territories. Several factors were identified in this study responsible for malaria resurgence in Panamá, mostly related with Malaria Control Programme weakening. Data analysis confirmed that although authorities have been successful in focalizing malaria transmission in the country, there are still neglected issues to be solved and important intercultural barriers that need to be addressed in order to achieve elimination of the disease in Panama by 2022, as established by the Strategic Plan for the Elimination of Malaria.

## Supplementary information

**Additional file 1: Figure S1** Malaria cases in Panamá by Plasmodium species between 1957 and 2019.

**Additional file 2: Figure S2** Malaria in Panamá by Annual Parasite Index (API) between 1957 and 2018.

**Additional file 3: Figure S3** Autochthonous and imported malaria cases by Plasmodium species in Panamá, 2000–2019.

**Additional file 4: Table S1** Imported malaria cases in Panamá by country of origin, 2000 – 2019.

## Data Availability

The datasets analysed during the current study are available from the corresponding author on reasonable request.

## Abbreviations

API: Annual parasite incidence
CQ: Chloroquine
DDT: Dichloro-Diphenyl Tricloethane
ENSO: El Niño Southern Oscillation
ICGES: Instituto Conmemorativo Gorgas de Estudios de la Salud
IRS: Indoor Residual Spraying
MoH: Ministry of Health
MEP: Malaria Eradication Programme
NMCP: National Malaria Control Programme
NMEP: National Malaria Elimination Programme
PAHO: Pan American Health Organization
WHO: World Health Organization
