# Supplementary figures and images for: Long-term transmission patterns and public health policies leading to malaria elimination in Panamá
Source: Malar J. 2020 Jul 23;19:265. doi: 10.1186/s12936-020-03329-y (PMC7376851; doi:10.1186/s12936-020-03329-y)

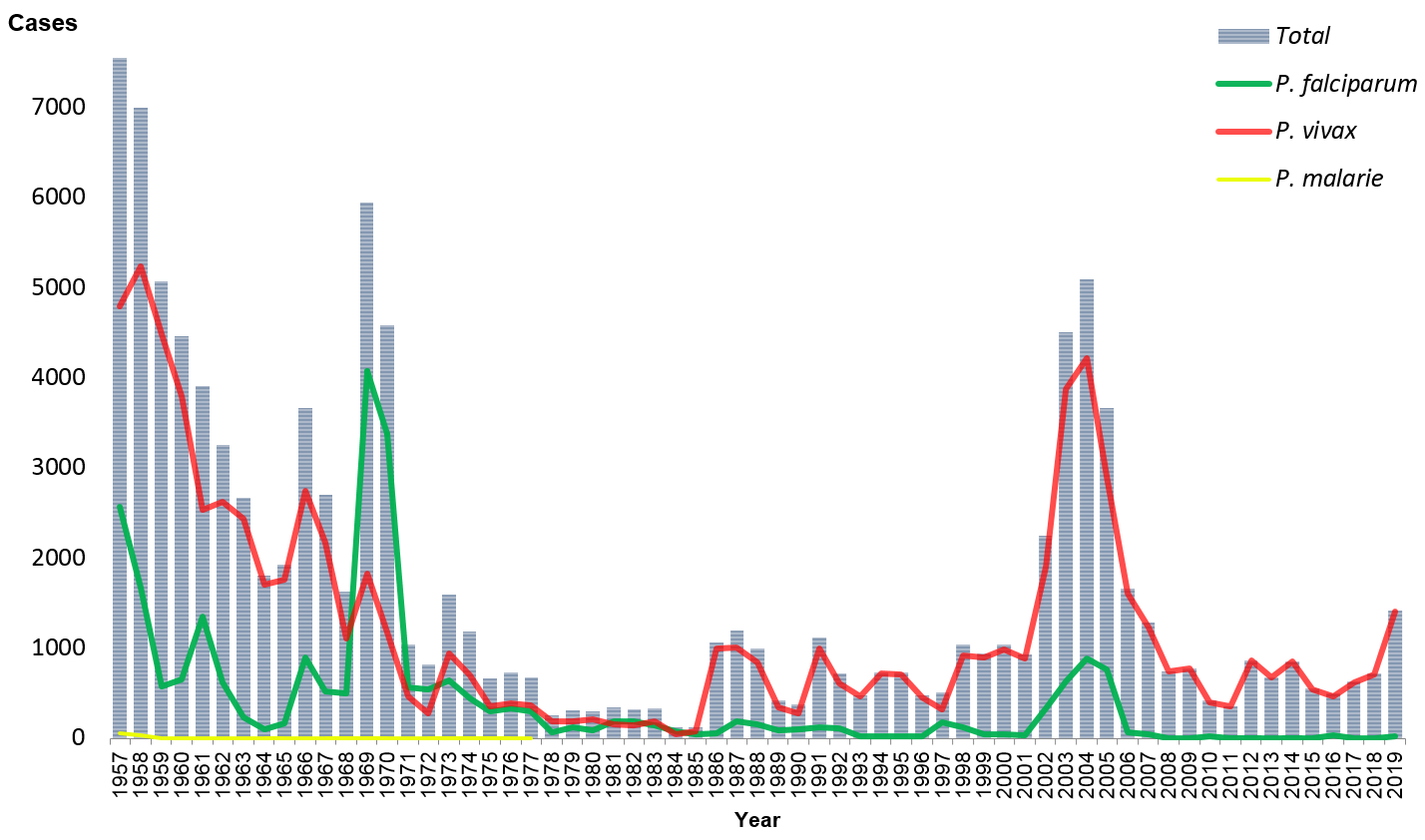

Supplement: Supplementary file 1 — Additional file 1: Figure S1 Malaria cases in Panamá by Plasmodium species between 1957 and 2019. [file 12936_2020_3329_MOESM1_ESM.tif]

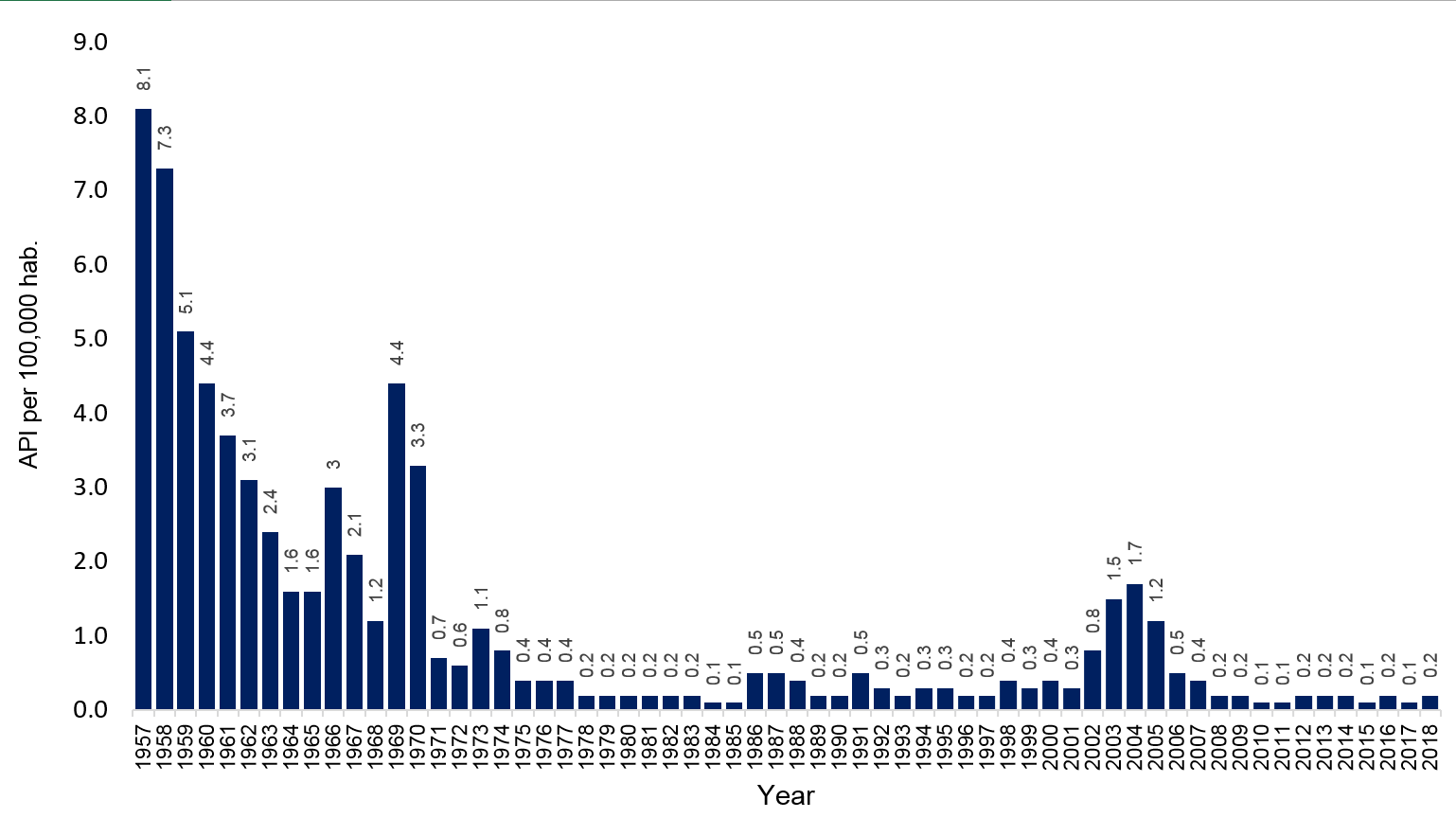

Supplement: Supplementary file 2 — Additional file 2: Figure S2 Malaria in Panamá by Annual Parasite Index (API) between 1957 and 2018. [file 12936_2020_3329_MOESM2_ESM.tif]

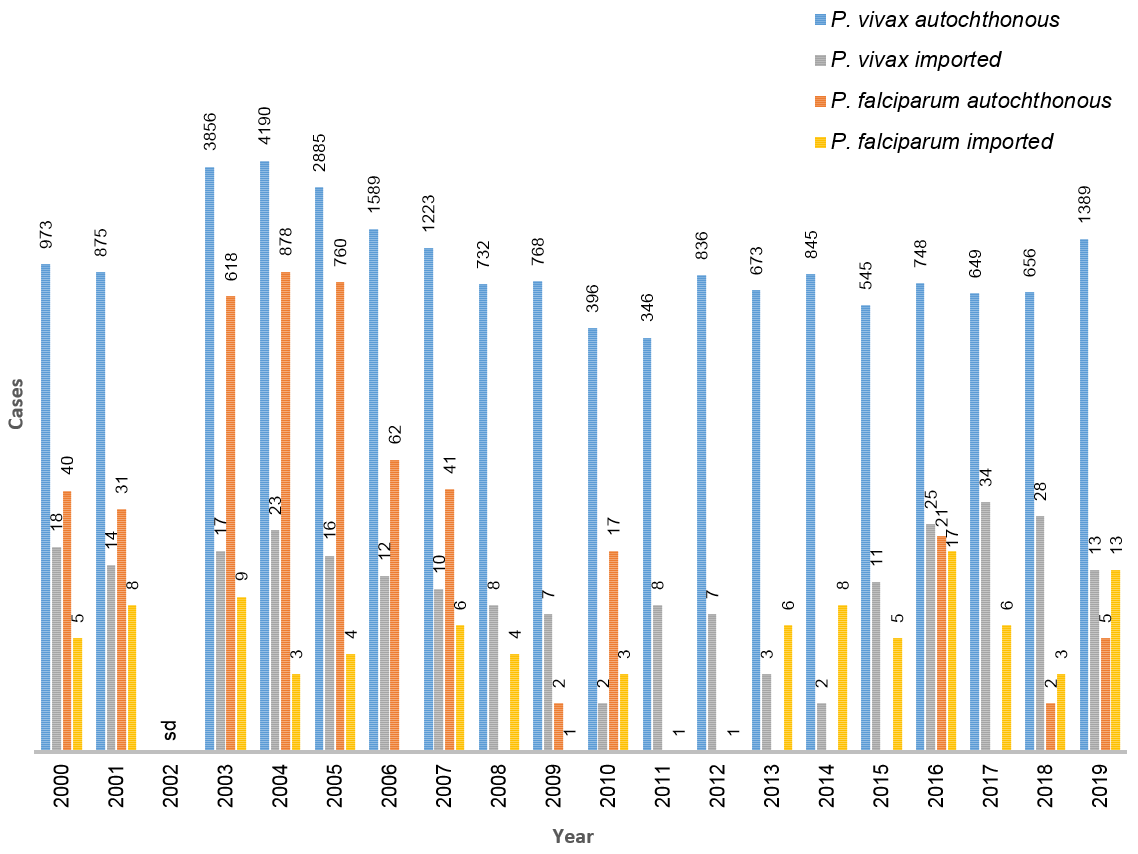

Supplement: Supplementary file 3 — Additional file 3: Figure S3 Autochthonous and imported malaria cases by Plasmodium species in Panamá, 2000–2019. [file 12936_2020_3329_MOESM3_ESM.tif]
