# Supplementary material for: Long-term transmission patterns and public health policies leading to malaria elimination in Panamá
Source: Malar J. 2020 Jul 23;19:265. doi: 10.1186/s12936-020-03329-y (PMC7376851; doi:10.1186/s12936-020-03329-y)
Supplement: Supplementary file 4 — Additional file 4: Table S1 Imported malaria cases in Panamá by country of origin, 2000 – 2019. [file 12936_2020_3329_MOESM4_ESM.docx]

| **Country** | **Total** | | ***P. falciparum*** | | ***P. vivax*** | |
| --- | --- | --- | --- | --- | --- | --- |
|  | **Frequency** | **Percentage** | **Frequency** | **Percentage** | **Frequency** | **Percentage** |
| **TOTAL** | **360** | **100** | **103** | **100.0** | **257** | **100.0** |
| Colombia | 175 | 48.6 | 56 | 54.4 | 119 | 46.3 |
| Costa Rica | 68 | 18.9 | 0 | 0.0 | 68 | 26.5 |
| Africa | 47 | 13.1 | 40 | 3.8 | 7 | 2.7 |
| Nicaragua | 14 | 3.9 | 0 | 0.0 | 14 | 5.4 |
| India | 14 | 3.9 | 2 | 1.9 | 12 | 4.7 |
| Venezuela | 13 | 3.6 | 0 | 0.0 | 13 | 5.1 |
| Honduras | 9 | 2.5 | 2 | 1.9 | 7 | 2.7 |
| Peru | 4 | 1.1 | 0 | 0.0 | 4 | 1.6 |
| Cuba | 3 | 0.8 | 0 | 0.0 | 3 | 1.2 |
| Brazil | 3 | 0.8 | 0 | 0.0 | 3 | 1.2 |
| Guyana | 1 | 0.3 | 0 | 0.0 | 1 | 0.4 |
| Egypt | 1 | 0.3 | 1 | 1.0 | 0 | 0.0 |
| Pakistan | 1 | 0.3 | 0 | 0.0 | 1 | 0.4 |
| Haiti | 1 | 0.3 | 1 | 1.0 | 0 | 0.0 |
| Dominican Republic | 1 | 0.3 | 0 | 0.0 | 1 | 0.4 |
| Trinidad and Tobago | 1 | 0.3 | 0 | 0.0 | 1 | 0.4 |
| Guatemala | 1 | 0.3 | 0 | 0.0 | 1 | 0.4 |
| Japan | 1 | 0.3 | 0 | 0.0 | 1 | 0.4 |
| Italy | 1 | 0.3 | 0 | 0.0 | 1 | 0.4 |
| Philippines | 1 | 0.3 | 1 | 1.0 | 0 | 0.0 |
| *Data until epidemic week 45 of 2019 | | |  |  |  |  |

**Additional file 4**. Imported malaria cases in Panamá by country of origin, 2000 – 2019
